# Folic Acid Reduces Tau Phosphorylation by Regulating PP2A Methylation in Streptozotocin-Induced Diabetic Mice

**DOI:** 10.3390/ijms18040861

**Published:** 2017-04-19

**Authors:** Miaoyan Zheng, Chen Zou, Mengyue Li, Guowei Huang, Yuxia Gao, Huan Liu

**Affiliations:** 1 Key Laboratory of Hormones and Development (Ministry of Health), Tianjin Key Laboratory of Metabolic Diseases, Tianjin Metabolic Diseases Hospital & Tianjin Institute of Endocrinology, Tianjin Medical University, Tianjin 300070, China; myzheng73@163.com; 2 Department of Nutrition and Food Science, School of Public Health, Tianjin Medical University, Tianjin 300070, China; zouchen_0709@163.com (C.Z.); limengyue0410@163.com (M.L.); huangguowei@tmu.edu.cn (G.H.); 3 Department of Nutrition, Tianjin Stomatological Hospital, Tianjin 300041, China; 4 Department of Cardiology, General Hospital of Tianjin Medical University, Tianjin 300052, China; gaoyuxia@medmail.com.cn

**Keywords:** folic acid, diabetes, tau phosphorylation, PP2A, methylation

## Abstract

High incidence rate of Alzheimer’s disease (AD) is observed in patients with type 2 diabetes. Aggregated β-amyloid (Aβ) and hyperphosphorylated tau are the hallmarks of AD. Hyperphosphorylated tau has been detected in diabetic animals as well as in diabetic patients. Folates mediate the transfer of one carbon unit, required in various biochemical reactions. The effect of folate on tau phosphorylation in diabetic models still remains unknown. In this study, we investigated the effect and mechanism of folic acid on hyperphosphorylation of tau in streptozotocin (STZ)-induced diabetic mice. Diabetic mice induced by STZ, at the age of 10 weeks, were administered with three levels of folic acid: folic acid-deficient diet, diet with normal folic acid content, and 120 μg/kg folic acid diet for 8 weeks. Levels of serum folate and blood glucose were monitored. Tau phosphorylation, protein phosphatase 2A (PP2A) methylation, and Glycogen synthase kinase 3β (GSK-3β) phosphorylation were detected using Western blot. The S-adenosyl methionine:S-adenosyl homocysteine ratio (SAM:SAH) in brain tissues was also determined. DNA methyltransferase (DNMT) mRNA expression levels were detected using real-time PCR. Folic acid reduced tau hyperphosphorylation at Ser396 in the brain of diabetes mellitus (DM) mice. In addition, PP2A methylation and DNMT1 mRNA expression were significantly increased in DM mice post folic acid treatment. GSK-3β phosphorylation was not regulated by folic acid administration. Folic acid can reduce tau phosphorylation by regulating PP2A methylation in diabetic mice. These results support that folic acid can serve as a multitarget neuronal therapeutic agent for treating diabetes-associated cognitive dysfunction.

## 1. Introduction

Epidemiological studies show high incidence rate of Alzheimer’s disease (AD) in patients with type 2 diabetes [1,2,3]. A number of cross-sectional and longitudinal studies have examined the association between type II diabetes mellitus (DM) and cognitive impairment [4,5], mild cognitive impairment (MCI), and dementia [6]. Extracellular senile plaques (SP) and intracellular neurofibrillary tangles (NFTs) are the hallmarks of AD. Amyloid-β (Aβ), a peptide derived from the amyloid protein precursor (APP), and hyperphosphorylated tau are the major components of senile plaques and NFTs respectively [7].

Tau is a neuronal cytoskeletal protein responsible for microtubulin polymerization and stabilization. It suggests that tau, a microtubule associated protein, also plays a key role in the pathogenesis of AD as Aβ. Aberrantly hyperphosphorylated tau fails to bind and stabilize microtubules, resulting in destabilization of the cytoskeleton and perturbation of axonal transport [8]. Hyperphosphorylated tau has been previously detected in brain of experimental diabetic animal [9,10] as well as in diabetic patients [11,12]. Furthermore, phosphorylation of tau can be triggered by high glucose in hippocampal neurons [13]. It can be seen that hyperphosphorylated tau is one of the common pathological changes of both AD and DM.

Protein phosphatase 2A (PP2A) is the major brain serine/threonine protein phosphatase that can dephosphorylate tau at multiple sites. Post-mortem AD brains had reduced PP2A expression and activity while inhibition of PP2A resulted in AD-like tau pathology and cognitive impairment in animal models [14,15,16]. Factors affecting PP2A amount and activity are still emerging; however, at least two mechanisms are known to be involved: methylation and phosphorylation [17,18].

PP2A is composed of A, B and C subunits. Methylation of subunit C at Leu-309 by means of a specific S-adenosylmethionine (SAM)-dependent methyltransferase affects the substrate specificity of it. PP2A might link tau phosphorylation to homocysteine (Hcy) and SAM metabolism [19,20]. This suggests a possible effect of dietary factors related to one carbon unit metabolism on the process of neurodegeneration.

Diabetes can induce perturbations of methyl group and homocysteine metabolism. Plasma hcy concentration was found higher in DM patients [21]. Genomic DNA was hypomethylated in liver of diabetic rats [22]. Folate mediates the transfer of one carbon unit required in various biochemical reactions. It plays a critical role in the synthesis of SAM, which serves as the methyl group donor in several methylation reactions involving DNA, RNA, and protein [23]. Folic acid supplementation in patient with type 2 DM can reduce Hcy levels and have a trend to associate with better glycemic control compared with placebo [24]. So, we hypothesized that folic acid may reduce tau phosphorylation by regulating methyl metabolism under diabetic conditions.

We had previously demonstrated that folic acid could downregulate tau protein phosphorylation by inhibiting the demethylation reactions of PP2A in SH-SY5Y cells [25]. Nicolia et al. demonstrated that Glycogen synthase kinase 3β (GSK-3β) and PP2A genes were upregulated by vitamin B deficiency-mediated inhibition of methylation reactions [18]. Sontag et al. indicated folate-deficient diets have brain-region-specific alterations in PP2A methylation in mice [26]. In the present study, we observed the effect of folic acid deficiency and supplement on tau phosphorylation and PP2A methylation in mice with DM.

## 2. Results

### 2.1. Serum Folate and Blood Glucose

**Figure 1:**
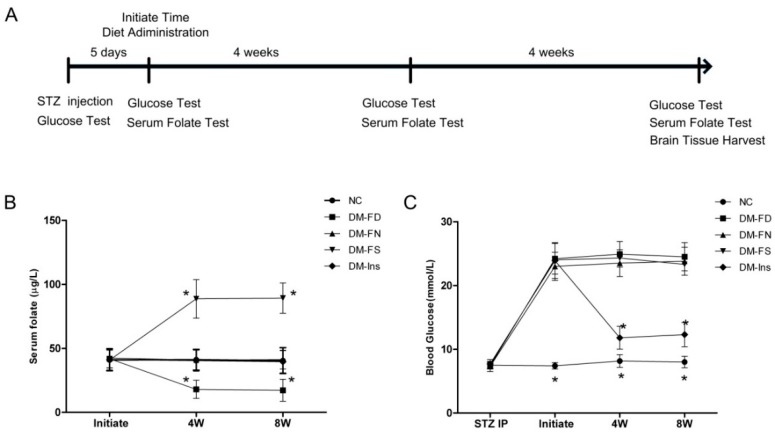
Experimental timeline and results of serum folate and blood glucose level. Timeline depicting serum folate as well as blood glucose testing at baseline and every 4 weeks throughout the study (**A**); STZ-treated mice were divided into four groups: folate-deficient diet plus daily gavage with water (DM-FD); control diet plus daily gavage with water (DM-FN); control diet plus daily gavage with 120 μg/kg folic acid (DM-FS) and control diet plus daily gavage with water and insulin treatment (DM-Ins). Additionally, mice fed with the control diet and gavaged daily with water as normal control group (NC). Serum folate in STZ-treated mice was determined by an automated chemiluminescence system (**B**); Blood glucose in tail vein was tested by glucometer (**C**). Plotted are mean ± SD values, *n* = 11 animals/group. * *p* < 0.05 versus the DM-FN group. STZ, streptozotocin.

Post induction of diabetes in mice, the serum folate level was detected thrice during folate or insulin treatment. As shown in Figure 1, DM mice, fed with the folate-deficient diet (DM-FD), had lower serum folate concentrations (*p* < 0.05) than those fed with the control diet (DM-FN). On the contrary, folic acid administration in DM mice (DM-FS) increased serum folate levels (*p* < 0.05). Insulin injection could reduce blood glucose significantly but not influence serum folate level. Folate deficiency or supplementation did not affect the blood glucose level during the entire experimental period.

### 2.2. Effect of Folic Acid on TAU Phosphorylation in DM Mice

**Figure 2:**
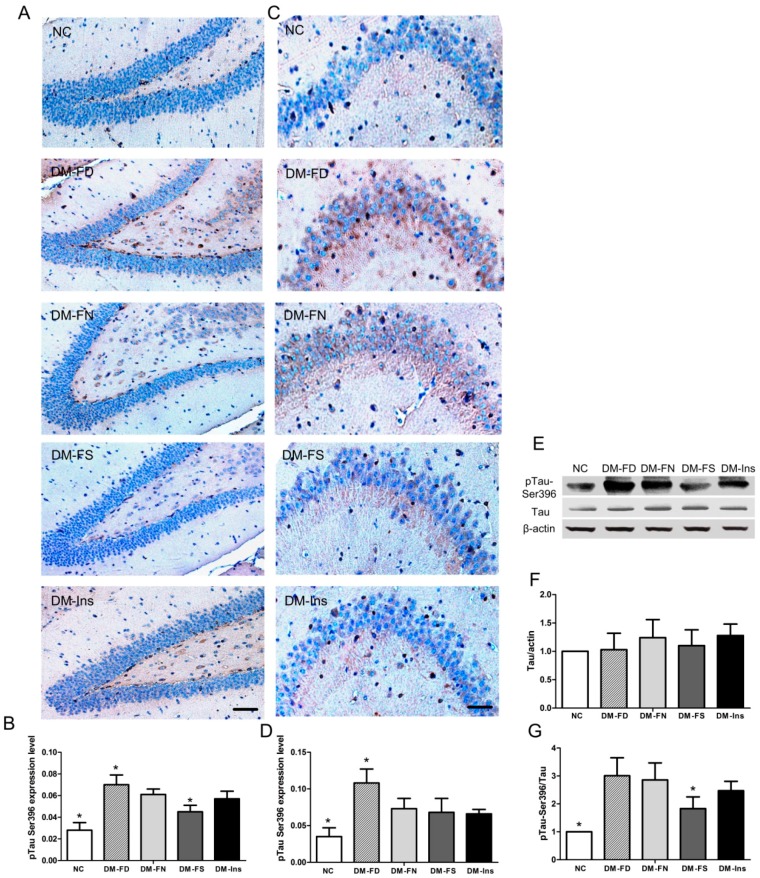
Impact of folic acid on tau phosphorylation in STZ-treated mouse brains. Immunohistochemistry of phosphorylated tau at epitopes Ser 396 in STZ-treated (or Control) mouse brains in CA3 (**A**,**B**) and DG area of hippocampus (**C**,**D**). Western blot analysis (**E**) and quantification of total tau (**F**) and phosphorylated tau at epitopes Ser 396 (**G**) in STZ-treated (or Control) mouse brains. The data were expressed as mean ± SD values, *n* = 5 animals/group. * *p* < 0.05 versus the DM-FN group. Scale bar = 50 μm (**A**); Scale bar = 20 μm (**C**).

Besides Aβ deposition, abnormally phosphorylated tau is a major neuropathological characteristic of AD. In the present study, tau hyperphosphorylation was assessed by Western blot and immunohistochemistry using antibodies against phosphorylated site (Ser396) on tau. As shown in Figure 2, there was significant increase in tau phosphorylation at sites of Ser396 in both the hippocampal regions, CA3 and DG, in DM mice fed normal diet compared to that in the control group. Folate deficiency further enhanced tau phosphorylation in both regions. Folate supplementation inhibited tau phosphorylation only in the hippocampal DG region. No significant difference was observed for the pTau Ser396 expression level in the hippocampal area, CA3 between the DM-FS and DM-FN groups.

Quantitative Western blot analysis of brain tissue of the DM mice got an increase in tau phosphorylation compared to the normal mice. Administration with folate revealed a significant decrease in tau phosphorylation compared to that in the DM mice fed with control diet. However, the pTau Ser396 expression level in DM-FD group exhibited an increasing trend, though statistically non-significant. Similarly, insulin treatment showed a decreasing trend on pTau Ser396 expression but statistically non-significant.

### 2.3. Effect of Folic Acid on GSK-3β Phosphorylation

**Figure 3:**
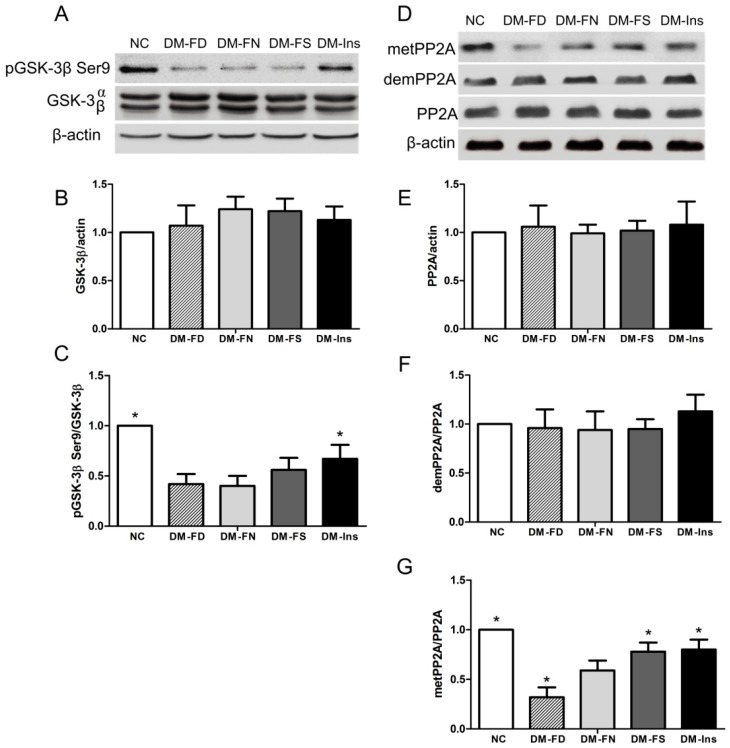
Effect of folic acid on GSK-3β and PP2A peptides in STZ-treated mouse brains. Representative Western blot image of p-GSK-3β (Ser9), GSK-3β and β-actin respectively (**A**); Quantified results of GSK-3β was normalized to β-actin (**B**); Relative expression level of p-GSK-3β (Ser9) was normalized to GSK-3β expression (**C**); Representative Western blot image of anti-methylated, anti-demethylated and total PP2A (**D**); histograms showing semi-quantitative analyses of anti-methylated (**E**); anti-demethylated (**F**) and total PP2A (**G**). The data were expressed as means ± SD values, *n* = 5 animals/group. * *p* < 0.05 versus the DM-FN group. PP2A, protein phosphatase 2A; GSK-3β, glycogen synthase kinase 3β.

Among several kinases involved in tau phosphorylation, GSK-3β has been implicated to play the most important role in its abnormal hyperphosphorylation. As shown in Figure 3, the western bolt result revealed that STZ treatment inhibited GSK-3β phosphorylation compared to normal mice. Folate deficiency or supplementation did not affect GSK-3β phosphorylation at Ser9 in STZ-induced diabetic mice. GSK-3β phosphorylation was significantly high in DM-Ins group compared to that in the DM-FN group.

### 2.4. Effect of Folic Acid on PP2A Methylation

PP2A dysfunction has been linked to tau hyperphosphorylation, amyloidogenesis, and synaptic deficits that are pathological hallmarks of AD. Deregulation of PP2A enzyme also affects the activity of many Ser/Thr protein kinases implicated in AD.

Quantitative Western blot analysis of mouse brain tissue extracts revealed that DM model induced by STZ treatment decreased PP2A methylation compared to normal mice. Meanwhile, folate deficiency inhibited PP2A methylation compared to DM mice fed with the control diet (Figure 3). Both folate supplementation and insulin injection stimulated PP2A methylation but not its expression.

### 2.5. Methylation Potential

**Figure 4:**
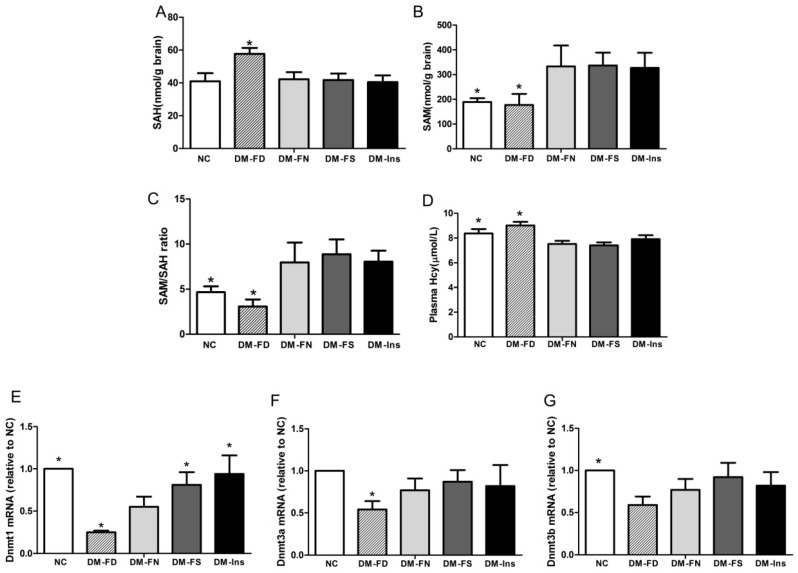
Influence of folic acid on methyl potential and DNMT mRNAs expression in STZ-treated mouse brains. Concentration of brain SAH (**A**), SAM (**B**) and plasma HCY (**D**) were determined by HPLC. SAM:SAH ratio (**C**) is generally considered to be a good indicator of DNA methylation potential. Gene expression of DNMT1 (**E**), DNMT3a (**F**) and DNMT3b (**G**) were measured by real-time PCR. The data were expressed as means ± SD values, *n* = 6 animals/group. * *p* < 0.05 versus the DM-FN group. DNMT, DNA methyltransferase; SAM, S-adenosyl methionine; SAH, S-adenosyl homocysteine; HCY, homocysteine.

We determined whether the effects of folic acid deficiency or supplementation on tau phosphorylation were accompanied by changes in the methylation potential. As shown in Figure 4, folate deficiency increased SAH concentration and decreased SAM level, thereby reducing methylation potential (SAM:SAH ratio) in the brain tissue of DM mice. On the contrary, folate deficiency resulted in a significant increase in the concentration of plasma Hcy. However, neither folic acid supplementation nor insulin treatment significantly affected the methylation potential and plasma Hcy.

### 2.6. Effect of Folic Acid on DNMT mRNA Expression

DNMT gene expression was studied using real-time PCR (RT-PCR). The results demonstrated a significant increase in the levels of DNMT1 mRNA in the brains of DM mice administrated with folic acid or insulin compared to that in the DM + FN group. Folate-deficient diet caused a significant decrease in DNMT1 and DNMT3a mRNA expression in the brains of STZ-treated mice. DNMT3b level did not show significant variation in the DM mice of the four groups.

## 3. Discussion

Epidemiological studies strongly suggest that diabetes mellitus is a risk factor for AD [18,19], although the causal relationship remains poorly understood. In an 11-year follow-up study of a Taiwanese population, diabetic patients were found to be more susceptible to AD compared to non-diabetic patients [27]. The link between AD and diabetes has been supported by studies using mouse models [28,29]. Pathological features of AD, including tau phosphorylation and amyloid plaque deposition, were exaggerated in the brains of APP transgenic mice following the induction of insulin-deficient diabetes [28].

STZ is a diabetogenic substance used in diabetes research to induce insulin depletion after intraperitoneal injection. Recent study indicated that type 1 DM [30] model induced by STZ as well as spontaneous type 2 DM [31,32] model showed overexpression of phosphorylated tau at Ser396 cite. In the present study, we also found tau hyperphosphorylation at Ser396 in hippocampus after STZ treatment in mice fed with the control diet, which further confirmed that insulin deficiency caused by STZ could increase phosphorylation of tau.

GSK-3β and PP2A can both modify tau phosphorylation. Overexpression of GSK-3β can enhance tau hyperphosphorylation and the formation of neurofibrillary tangles in the brain [33]. Down-regulation of PP2A is partially responsible for the abnormal tau phosphorylation in the brain. In the present study, phosphorylation of GSK-3β at Ser9 was significantly decreased by insulin deficiency after STZ injection, indicating increased GSK-3β activity. On the other hand, GSK-3β phosphorylation was enhanced by insulin treatment. These findings support that diabetes increases the risk for developing AD through the impairment of insulin signaling that leads to over-activation of GSK-3β and, consequently, abnormal hyperphosphorylation of tau [34]. Increasing expression of demPP2A has been found in type 1 DM mice [35]. In our study, metPP2A was downregulated by STZ treatment, which also indicates diabetes leading to a progressive AD-like tau hyperphosphorylation by regulating PP2A methylation. The mechanism of low methylation status of PP2A in DM brains may be due to an increasing requirement for methyl donors in brain tissue under diabetic status [36].

Folate is essential for transfer one-carbon units required for the synthesis of DNA/RNA and the methyl groups required to regenerate methionine from homocysteine. Several studies have investigated the relationship between DNA methylation with respect to type 2 diabetes [37]. We had previously shown that folic acid could inhibit tau phosphorylation through regulating PP2A methylation in vitro. In the present study, we aimed to investigate whether folic acid could affect tau phosphorylation by modifying the major kinase and phosphates involved in this process and methyl potential in diabetic mice brain.

During the eight weeks of our study, diets deficient and supplemented with folic acid lowered and increased serum folate levels, respectively. Blood glucose was not regulated by folic acid administration. Meanwhile, no serum folate changed by STZ and insulin treatment. Immunohistochemistry results demonstrated that folate deficiency further enhanced tau phosphorylation at Ser396 in both CA3 and DG hippocampal regions induced by STZ injection. Folic acid supplementation inhibited tau phosphorylation only in the CA3 region. Similar trends were seen in the Western blot results.

Few studies explored the effect of folic acid on GSK-3β; some of them indicated that folic acid could regulate GSK-3β Ser9 phosphorylation or GSK-3β expression in model of cardiac teratogenicity [38] or patients with colorectal adenomas [39]. However, in the present experiment, we did not observe a significant difference in GSK-3β phosphorylation nor GSK-3β expression among the DM-FD, DM-FN, and DM-FS groups.

In the present study, we analyzed PP2A methylation among DM mice, subjected to treatment with different levels of folic acid. Folic acid deficiency induced a decrease in methylated PP2A levels in the brain of DM mice. Similar downregulation of PP2A methylation and concomitant phosphorylation of tau and/or APP in the brain of AD mice caused by dietary folate and B-vitamin deficiency [17,26] and elevated homocysteine levels [20] have been shown previously. In the present study, folic acid supplementation caused an increase in methylated PP2A expression, as observed in our previous in vitro study. Those results supported that folic acid may serve as a neuronal therapeutic agent for treating diabetes-associated cognitive dysfunction.

The methylation cycle metabolites and DNMT expression in the brain tissue of DM mice confirmed the effect of STZ and folic acid on PP2A methylation. A meta-analysis revealed that elevated Hcy level was causally associated with the increased risk of DM [40]. However, lower plasma Hcy concentration has been recorded in animal models of DM [22,41]. In the present study, STZ injection induced hypohomocysteinemia; an observation reported in previous works as well [42,43]. That may be the result of increasing folate-independent remethylation of homocysteine by betaine-homocysteine *S*-methyltransferase (BHMT), which is upregulated in DM status [41].

Folate or other B vitamins—such as vitamins B6 and B12—are necessary for proper functioning of one-carbon metabolism pathway [23]. In the present study, folate deficiency restricted Hcy metabolism, resulting in brain and plasma SAH accumulation in STZ-treated mice. However, folic acid supplementation did not result in a significant increase of the SAM:SAH ratio in comparison to the group fed with diet comprising normal levels of folic acid. It seems that excessive supplementation of folic acid may be unnecessary for optimal maintenance of the methylation cycle [38].

DNMT3a and DNMT3b establish DNA methylation patterns, and DNMT1 subsequently maintains it. Expression levels of DNMTs mRNA were downregulated in the STZ-treated mice in our study. The possible reason for abnormal maintaining of DNA methylation in DM mice may be related to more consuming of methyl donors in the process of phosphatidylcholine biosynthesis, which is regulated by phosphatidylethanolamine methyl transferase (PEMT) in diabetic mice brain [36]. Folic acid deficiency further downregulated DNMT1 and DNMT3a mRNA levels in DM mice. This may be associated with the increasing level of SAH, which is an inhibitor of DNA methylation. The effect of folic acid on DNMT expression varied with different models of diseases. For example, folic acid increased DNMT1 expression in breast cancer cell lines [44]. However, hepatic DNMT1 mRNA and protein expression were elevated by folate-deficient diet in rat [45]. In our study, folic acid administration rescued the expression of DNMT1 mRNA, which was downregulated by STZ treatment. The mechanism of folic acid on regulating DNMTs is still unclear and needs further investigation.

## 4. Materials and Methods

### 4.1. Animals and STZ Treatment

All study procedures were performed in accordance with protocol approved by the Tianjin Medical University Animal Ethics Committee (permission code: TMUaMEC 2012016; permission date: 20120506); 9–10-week-old male C57Bl/6J mice were purchased from Beijing HFK Bioscience Co., Ltd. (Beijing, China). The animals were housed in the Animal Housing Facility of Tianjin Medical University using polycarbonate cages with paddy husk bedding in the animal room. The room temperature and relative humidity were maintained at 22 ± 2 °C and 55 ± 10%, respectively, with a 12 h light/dark cycle.

To induce diabetes in the mice, which were kept in fasted condition for 12 h prior to injection, a single intraperitoneal injection of 150 mg STZ (Sigma Chemical Co., St. Louis, MO, USA), prepared in cold citrate buffer (0.1 M; pH 4.5), was administered per kg of mouse body weight. The same volume of citrate buffer was injected into the control group, instead of STZ.

Blood was obtained from the tail vein of each mouse five days post-STZ injection. Blood glucose levels were monitored using a glucose meter. The mice with blood glucose level above 16.7 mmol/L were considered to be diabetic and used for the subsequent experiments.

### 4.2. Experimental Diets

In total, 44 mice, successfully induced with diabetes, were divided into four groups: (1) folate-deficient diet plus daily gavage with water (DM-FD, *n* = 11); (2) control diet (normal folic acid content) plus daily gavage with water (DM-FN, *n* = 11); (3) control diet plus daily gavage with 120 μg/kg folic acid (DM-FS, *n* = 11); and (4) control diet plus daily gavage with water and insulin treatment (DM-Ins, *n* = 11). In the DM-Ins group, an injection with dose adjustments (2–3 units per kg mouse body weight of Glargine Insulin) was administered once daily to maintain the level of blood glucose within 10–16 mmol/L. In addition, the control mice were fed with the control diet and gavaged daily with water (NC, *n* = 11).

The folate-deficient diet (0.2 mg/kg folic acid diet) and the control diet (2.1 mg/kg folic acid diet) were purchased from Test Diet (St. Louis, MO, USA). All the mice received food and drinking water ad libitum. Diets were administered for eight weeks.

All mice were sacrificed by cervical dislocation. In order to assess the status of tau phosphorylation, the right half of the brain from five mice of each group was used for Western blotting, while the left half was used for immunohistochemistry of the phosphorylated tau protein. The right half of the brain from the other six mice of each group was used for real-time PCR, whereas the left half was used to perform HPLC for the evaluation of methyl potential.

### 4.3. Serum Folate, Blood Glucose, and Plasma Hcy Levels

Serum folate levels were determined five days after STZ treatment and then every four weeks, using an automated chemiluminescence system (Immulite 1000; Siemens, Berlin, Germany).

Tail vein blood glucose level was monitored by using a glucometer (Accu-Chek; Roche, Penzberg, Germany). Blood glucose test strips were used to measure the blood glucose concentration in mice.

Plasma levels of Hcy were analyzed using high-performance liquid chromatography (HPLC) with a Waters 700 HPLC Pump and a reversed-phase C18 column (5 µm bead size; 4.6 mm × 250 mm) (Waters, Milford, CT, USA). Briefly, the mobile phase consisted of 0.08 M acetate buffer and 5% (*v*/*v*) methanol adjusted to pH 4.0 by addition of concentrated acetic acid and then filtered through a 0.45 µm membrane filter. The isocratic elution was performed using a flow rate of 1.0 mL/min at 30 °C and a pressure of 100–110 kgf/cm^2^ (1500–1800 psi). A fluorescence detector with excitation at 390 nm and emission at 470 nm was used to detect Hcy. Before analysis of Hcy, the system was calibrated with authentic DL-homocysteine standards in the range of 50 to 4000 ng. Plasma Hcy was quantified relative to the standard obtained from Sigma Chemical Co. (St. Louis, MO, USA).

### 4.4. Methylation Potential Assay

SAM, SAH, and the SAM:SAH ratio were determined in brain tissue samples. The brain tissue samples were homogenized using a motor-driven tissue homogenizer (PT1200E, Kinematica, Lucerne, Switzerland). Next, 100 mg extracts of the brain tissue were resuspended in 300 μL 0.4 mol/L perchloric acid. Homogenates were centrifuged at 20,000× *g* for 10 min at 4 °C. The supernatant was filtered through a 0.45-μm membrane filter (Millipore, Billerica, MA, USA), followed by loading into a Venusil MP-C18 column (4.6 mm × 250 mm, 5 μm particle, Agela Technologies, Wilmington, DE, USA) fitted with a matched guard column, run by HPLC system (Waters, Milford, MA, USA). Absorption of eluted compounds was monitored at *λ* = 254 nm with an ultraviolet detector. A two-buffer elution system was used: mobile phase A and B, both contained 4 mmol/L 1-heptanesulfonic acid (pH 4) and 10 mmol/L ammonium formate. The mobile phase B contained 50% acentonitrile by volume. Elution of SAM and SAH was achieved at a flow rate of 1 mL/min with the following parameters: 0–0.5 min, 100% A; 0.5–20 min, linear gradient to 75% A and 25% B; 20–30 min, 25% B; 30–45 min, 100% A.

### 4.5. Real-Time PCR

Total RNA was extracted by Trizol. First-strand cDNA was synthesized from 1 μg total RNA. A 20-μL reaction volume was incubated sequentially for 50 min at 42 °C, 5 min at 90 °C, 5 min at 5 °C. Real-time PCR was performed using the LightCycler 480 SYBR Green I Master Kit (Roche, Mannheim, Germany). The 20-μL PCR mixture included 10 μL of PCR Master mix, 2 μL of cDNA, 1 μL of forward primer, 1 μL of reverse primer and 6 μL of water. The reaction mixtures were incubated at 95 °C for 5 min, followed by 45 amplification cycles (denaturation at 95 °C for 10 s; annealing at 61 °C for 10 s; extension at 72 °C for 10 s). Primers were specific for DNMT1 (forward, CCTAGTTCCGTGGCTACGAGGAGAA; reverse, TCTCTCTCCTCTGCAGCCGACTCA), DNMT3a (forward, GCCGAATTGTGTCTTGGTGGATGACA; reverse, CCTGGTGGAATGCACTGCAGAAGGA), DNMT 3b (forward, TTCAGTGACCAGTCCTCAGACACGAA; reverse, TCAGAAGGCTGGAGACCTCCCTCTT). The assay was performed using the Roche LightCycler 480 sequence detector (Roche, Mannheim, Germany).

### 4.6. Western Blotting

The total protein was extracted from the brain tissues using a lysis buffer. Homogenates were centrifuged at 12,000× *g* for 5 min at 4 °C, followed by mixing of the supernatants with the loading buffer and boiling for 5 min. Protein concentrations in the supernatants were determined using bicinchoninic acid (BCA) protein assay kit (Thermo Scientific, Vantaa, Finland), using bovine serum albumin (BSA) as a standard. Equal amounts of protein were loaded in each well for 10% sodium dodecyl sulfate-polyacrylamide gel electrophoresis and then the resolved proteins were transferred onto nitrocellulose membranes. The membranes were blocked with either 5% non-fat milk or 5% BSA and incubated with primary antibodies (anti-tau, 1:1000, CST, Framingham, MA, USA; anti-pTau Ser396, 1:5000, Abcam, Cambridge, MA, USA; anti-PP2A (C subunit), 1:500, Millipore, Billerica, MA, USA; anti-demPP2A (C subunit), 1:5000, Millipore, Billerica, MA, USA; anti-metPP2A (C subunit), 1:500, Millipore, Billerica, MA, USA; anti-GSK-3α/β, 1:1000, CST; anti-Phospho-GSK-3β (Ser9), 1:1000, CST, Framingham, MA, USA) overnight at 4 °C. After washing with TBST, the membranes were incubated with horseradish peroxidase-conjugated secondary antibody (1:10,000 in TBST) for 2 h and detected by chemiluminescence. Quantitation of proteins was done by densitometric analysis using NIH Image software (version 1.61). The intensity of each protein band was normalized to the respective actin band (anti-β-actin, 1:5000, Abcam, Cambridge, MA, USA).

### 4.7. Immunohistochemistry

The brains were removed and post-fixed with 4% paraformaldehyde in 0.1 M phosphate buffer (pH 7.4) at 4 °C overnight. The brains were coronally cut into 4-μm-thick sections using a vibratome. Free-floating sections were incubated with 4% bovine serum albumin in PBS for 1 h. This was followed by incubation with monoclonal anti-pTau Ser396 protein antibody (TauPhospho S396, 1:1000, CST, Massachusetts, MA, USA) at 4 °C overnight. The sections were washed with PBS, reacted with biotinylated secondary antibodies (diluted 1:200 in PBS) and visualized using ABC Elite kit (Vector Laboratories, Burlingame, CA, USA). Imaging was carried out using a microscope (Olympus, Tokyo, Japan). The mean optical density (MOD) and total per area (TPA) of CA3 and DG area in hippocampus were determined with Image-Pro Plus 6.0. MOD/lg(255) × TPA was calculated to determine the expression level of pTau Ser396.

### 4.8. Statistical Analysis

Results were expressed as mean ± S.D. and analyzed using the SPSS 13.0 software package (SPSS Inc., Chicago, IL, USA). Data of serum folate and blood glucose were analyzed by one-way ANOVA with repeated measures. Other data were analyzed by a one-way ANOVA followed by a Student–Newman–Keuls test; *p*-values < 0.05 were considered to be statistically significant.

## 5. Conclusions

In conclusion, tau hyperphosphorylation at Ser396 was found in the brains of STZ-induced diabetic mice. Folic acid reduced phosphorylation of tau by regulating metPP2A expression. Changes of methyl donor and DNMT activity may explain the effect of folic acid on methylation of PP2A.

## Abbreviations

AD	Alzheimer’s disease
STZ	streptozotocin
SAM	S-adenosyl methionine
SAH	S-adenosyl homocysteine
DNMT	DNA methyltransferase
